# Ellagic Acid Prevents α-Synuclein Aggregation and Protects SH-SY5Y Cells from Aggregated α-Synuclein-Induced Toxicity via Suppression of Apoptosis and Activation of Autophagy

**DOI:** 10.3390/ijms222413398

**Published:** 2021-12-13

**Authors:** Mustafa T Ardah, Nabil Eid, Tohru Kitada, M. Emdadul Haque

**Affiliations:** 1 Department of Biochemistry and Molecular Biology, College of Medicine and Health Sciences, United Arab Emirates University (UAEU), Al Ain P.O. Box 17666, United Arab Emirates; Mustafa_Ardah@uaeu.ac.ae; 2 Department of Anatomy, College of Medicine and Health Sciences, United Arab Emirates University (UAEU), Al Ain P.O. Box 17666, United Arab Emirates; nabileidm@uaeu.ac.ae; 3 Otawa-Kagaku, Parkinson Clinic and Research, Kamakura 247-0061, Japan; tohrukitada@gmail.com

**Keywords:** Parkinson’s disease, α-synuclein, Ellagic acid, neurodegeneration

## Abstract

Parkinson’s disease (PD) is a neurodegenerative disease characterized by the loss of dopamine neurons and the deposition of misfolded proteins known as Lewy bodies (LBs), which contain α-synuclein (α-syn). The causes and molecular mechanisms of PD are not clearly understood to date. However, misfolded proteins, oxidative stress, and impaired autophagy are believed to play important roles in the pathogenesis of PD. Importantly, α-syn is considered a key player in the development of PD. The present study aimed to assess the role of Ellagic acid (EA), a polyphenol found in many fruits, on α-syn aggregation and toxicity. Using thioflavin and seeding polymerization assays, in addition to electron microscopy, we found that EA could dramatically reduce α-syn aggregation. Moreover, EA significantly mitigated the aggregated α-syn-induced toxicity in SH-SY5Y cells and thus enhanced their viability. Mechanistically, these cytoprotective effects of EA are mediated by the suppression of apoptotic proteins BAX and p53 and a concomitant increase in the anti-apoptotic protein, BCL-2. Interestingly, EA was able to activate autophagy in SH-SY5Y cells, as evidenced by normalized/enhanced expression of LC3-II, p62, and pAKT. Together, our findings suggest that EA may attenuate α-syn toxicity by preventing aggregation and improving viability by restoring autophagy and suppressing apoptosis.

## 1. Introduction

PD is the most common type of neurodegenerative disorder that affects voluntary movement. The pathological hallmarks of the disease are progressive death of dopamine-producing neurons in the substantia nigra compacta (SNc) and accumulation of protein aggregates known as LBs in surviving neurons [1]. The major constituent of LBs is α-syn. The molecular mechanisms underlying the disease are mostly unknown. However, substantial evidence suggests that misfolded protein stress, mitochondrial dysfunction, oxidative stress, neuroinflammation, and dysfunctional autophagy play important roles in the pathogenesis of PD. α-syn is a highly abundant presynaptic protein with 140 amino acids [2]. It is naturally present in the unfolded form; however, it can take on an alpha-helical structure when bound to the cell membrane. The physiological functions of this protein are not fully understood. Mutations or gene duplication/triplication of α-syn (SNCA) have been linked to the familial form of PD. Thus, α-syn is not only associated with sporadic PD but also with the familial form of PD. It has also been reported that increased levels of α-syn are toxic to neurons and they accelerate the development of PD pathology.

Indeed, recent scientific data suggest that α-syn is prone to forming oligomers under physiological conditions. Under pathological conditions, it is well-accepted that α-syn follows the amyloid pathways of aggregation, that is, it first forms soluble oligomers and then insoluble aggregates [3]. Soluble oligomer species of α-syn have been shown to be the most toxic component [4]. The role of insoluble α-syn in physiological conditions is not clear; however, it has been suggested to be a part of this mechanism. Therefore, efforts have been ongoing to find a remedy that blocks the formation of soluble oligomers or prevents the process of aggregate formation. Thus, finding active biological compounds that prevent α-syn oligomerization or aggregate formation will lead to the development of novel therapeutic interventions for PD.

Ellagic acid (EA) is a naturally occurring poly-phenolic compound found in many fruits, such as pomegranates, persimmons, and raspberries. The presence of four hydroxyl groups in its chemical structure are responsible for its antioxidant properties. Multiple lines of evidence suggest that EA has numerous therapeutic benefits, such as anti-inflammatory, neuroprotective, hepatoprotective, anti-diabetic, anti-cancer, and prevention of cardiovascular disease [5,6]. EA has been reported to prevent the generation of S-nitrosylation of protein disulfide isomerase (SNO-PDI) formation upon rotenone exposure in a cellular model of PD [7]. It is noteworthy that nitrosative stress mediated S-nitrosylation (SNO) of protein disulfide isomerase (PDI), which is a housekeeping oxidoreductase, and it has been implicated in the pathogenesis of sporadic PD and Alzheimer’s disease. Previous cell line studies have indicated that SNO-PDI formation incites synphilin-1 aggregation, a minor biomarker protein of Parkinson’s disease. It has been reported that SNO-PDI formation is linked to the aggregation of α-syn and it also provokes α-syn: synphilin-1 deposits (LBs-like debris) that are normally found in the PD brain [8,9,10].

Interestingly, pomegranate juice, which is a rich in ellagitannins (ETs), is believed to provide a broad range of health benefits. It has been reported that treatment with pomegranate juice contributes neuroprotection. The neuroprotection is supported by observing the postural stability improvement, enhancement of neuronal survival, mitigating the oxidative damage and preventing α-syn aggregation, increased mitochondrial aldehyde dehydrogenase activity, and keeping the anti-apoptotic BCL-xL protein at the control level [11].

Macroautophagy (autophagy) is an anti-apoptotic pathway for the clearance of damaged cellular components, accumulated lipids, and abnormal proteins, specifically upon exposure to stressors such as oxidative stress, endoplasmic reticulum stress, and nitrative stress. Autophagy is characterized by the formation of autophagosomal membranes, which sequester the cellular components producing LC3-II-mediated autophagosomes. The latter then fuse with lysosomes to form autolysosomes for cargo clearance by lysosomal cathepsins [12,13]. A growing body of evidence indicates that α-syn induces the excessive accumulation of autophagosomes in neuronal cells of PD and AD by interfering with the maturation of autophagosomes into autolysosomes. This dysfunctional or impaired autophagy induced by α-syn results in neuronal cell apoptosis [14,15,16]. In addition, p62 is a prototypic autophagy adaptor that interacts with LC3-II on autophagosomes, resulting in their maturation and fusion with lysosomes. p62 was found to be downregulated in various models of PD and AD, resulting in the accumulation of immature autophagosomes and impaired autophagy [17].

The above findings prompted us to test whether EA can prevent α-syn aggregation and aggregated α-syn-induced cellular toxicity. Our findings suggest that EA inhibits α-syn aggregation. It also prevents α-syn aggregation, reduces apoptosis, and enhances the viability of SH-SY5Y cells via activation of autophagy.

## 2. Results

### 2.1. EA Inhibits α-Syn Amyloid Fibrils’ Formation In Vitro

The accumulation of α-syn as LBs and degeneration of dopamine-producing neurons in the SNc are considered as the pathological hallmarks of PD. Thus, α-syn has been regarded as the key player in the progression of sporadic and familial forms of PD. Hence, we decided to examine whether EA has any role in inhibiting the generation of α-syn fibril. We incubated α-syn at a concentration of 25 µM with continuous shaking for 11 days at 37 °C, and then we determined fibril formation by measuring the intensity of Th-S fluorescence at different time points. To evaluate the efficacy, α-syn was incubated with EA at molar ratios of 1:1, 1:2, and 1:4 (molar ratio of α-syn to EA), accordingly.

**Figure 1:**
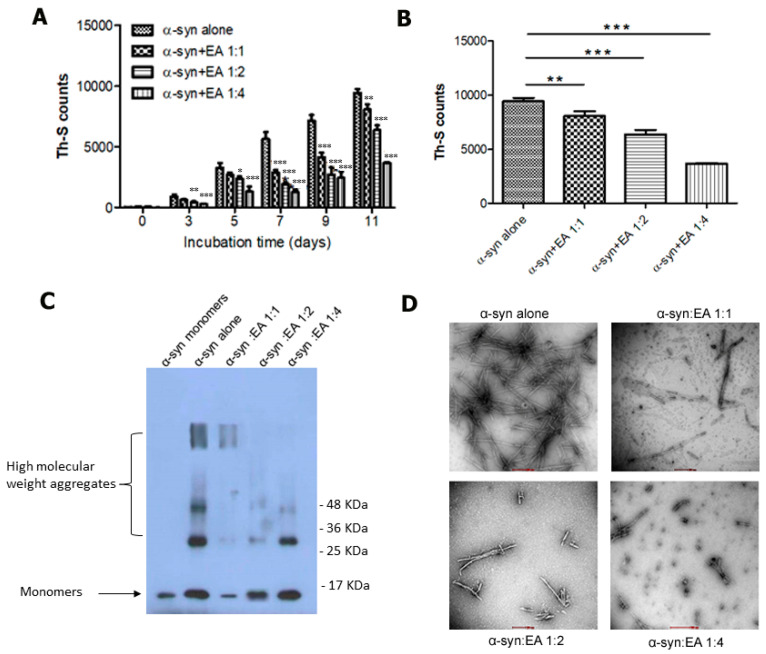
EA inhibits α-syn fibril formation in a concentration-dependent manner. 25 μM of α-syn samples were incubated in the presence or absence of EA in three different molar ratios (α-syn:EA, 1:1, 1:2, 1:4) at 37 °C with 800 rpm shaking for 11 days continuously. (**A**) Estimation of the fibril formation was conducted by the Th-S fluorescence assay. The assay was performed in triplicate and all values are mean ± standard deviation. (**B**) Th-S fluorescence assay results of day 11. (**C**) Western blot analysis to examine the effect of EA on α-syn fibrillation. Samples of α-syn incubated alone or with EA (α-syn:EA, 1:1, 1:2, 1:4) for 11 days (as described above) were analyzed by 15% SDS-PAGE gel and then probed against anti α-syn antibody (211). (**D**) Negatively stained electron microscopic images of the samples of α-syn:EA, 1:1, 1:2, 1:4, incubated for 11 days. Statistical analysis was performed using one-way ANOVA followed by the Bonferroni Multiple Comparison Test, ***, *p* < 0.001; **, *p* < 0.01; *, *p* < 0.05 compared with α-syn alone-treated group. Scale bar 500 nm.

Interestingly, we observed that EA inhibited α-syn aggregation, as indicated by reduced readout of Th-S fluorescence intensity (Figure 1A). More precisely, EA exhibited a significant inhibitory effect at the concentration of 100 µM, which was more remarkable on the fifth day of incubation. After 11 days of incubation (Figure 1B), EA at 100 µM decreased almost 80% fibrillation of α-syn, while at 50 µM it reduced fibrillation by almost 20%. At lower concentrations (i.e., 25 µM), EA also showed inhibition of α-syn fibrillation, the percentage of which was reduced by approximately 10% after nine days of incubation. Thus, these results indicated that EA prevented fibril formation in a dose-dependent manner.

To confirm the above findings, we performed additional tests, such as immunoblotting and electron microscopy. Briefly, the samples of α-syn were incubated alone or in the presence of EA at different molar ratios for 11 days and used in immunoblotting to confirm the results of the Th-S assay. Ten nanograms of samples were loaded and separated in 15% SDS gel, transferred to a PVDF membrane, and probed with anti α-syn monoclonal antibody 211 (Santa Cruz Biotechnology, Dallas, TX, USA). The radiographs (Figure 1C) clearly demonstrate the inhibition of the high molecular weight aggregates in the samples with the ratio of 4:1 (EA:α-syn molar ratio) compared to the aged α-syn sample alone, which supports the results obtained earlier. Transmission electron microscopic images of aged α-syn in the presence of EA showed different morphological features, dissimilar from the dense meshes of long fibrils formed by aged α-syn alone (Figure 1D), which again supports obtaining a reduced Th-S signal earlier.

### 2.2. Disaggregation of Preformed α-Syn Amyloid Fibrils by EA

**Figure 2:**
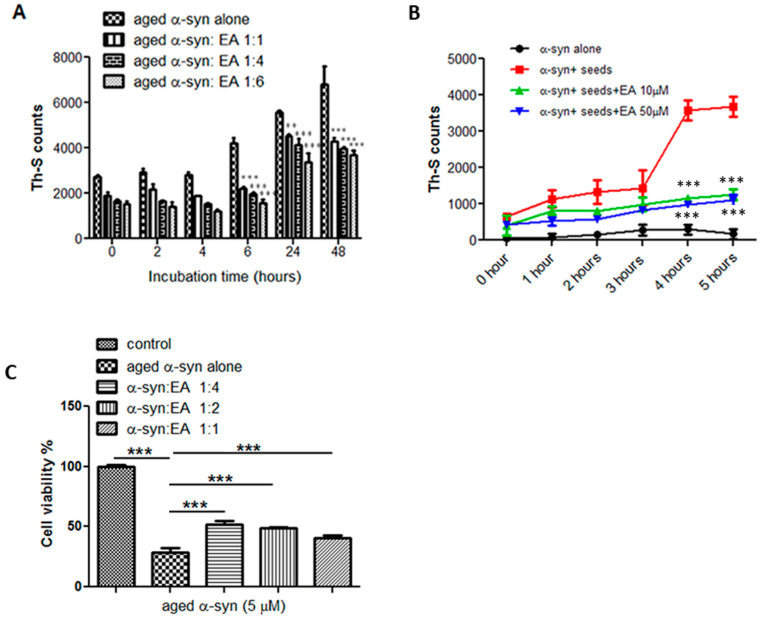
Preformed α-syn fibrils’ disaggregation by EA in a concentration-dependent manner, prevention of seeding aggregation and cell death. (**A**) Samples of preformed α-syn fibrils were incubated for 48 h at 37 °C with continuous shaking with or without EA at different molar ratios (aged α-syn:EA, 1:1, 1:4, 1:6). The fibril content was then estimated by the Th-S binding assay. The assays were performed in triplicate and all values are mean ± standard deviation. (**B**) α-syn monomers sample (100 μM) containing α-syn seeds (2 μM) incubated alone or in the presence of 10 or 50 μM EA for 5 h with continuous shaking at 37 °C. The amounts of preformed fibrils were estimated by the Th-S binding assay. The assays were performed in triplicate and all values are mean ± standard deviation. (**C**) The cytotoxicity of aged α-syn in SH-SY5Y human neuroblastoma cells was assessed by the MTT assay. The cells were treated for 48 h prior to MTT addition with samples of α-syn incubated alone or in the presence of EA (α-syn:EA, 1:1, 1:2, 1:4) for 11 days at 37 °C with continuous shaking. The final concentration of α-syn used for the MTT assay was 5 µM and EA at a concentration of 5, 10, or 20 µM (α-syn:EA, 1:1, 1:2, 1:4). The results are expressed as percentages of the control. Statistical analysis was performed using one-way ANOVA followed by the Bonferroni Multiple Comparison Test, ***, *p* < 0.001; **, *p* < 0.01 compared with aged α-syn alone-treated group for A and C, and with α-syn + seeds for B.

As shown before, that EA is considered an inhibitor of α-syn fibrillation, it was interesting to test whether EA can disaggregate the preformed fibrils and reverse the fibrillation process. To do this, preformed fibrils were prepared by incubating monomeric α-syn at 37 °C for 11 days with continuous shaking and a Th-S assay was performed to confirm fibril formation. Then, the prepared preformed α-syn fibrils at a concentration of 25 µM were incubated at 37 °C for 48 h in the presence of EA at molar ratios of 1:1, 1:4, and 1:6 of α-syn:EA. The fibril content was measured by Th-S fluorescence at different time points (0, 2, 4, 6, 24, and 48 h) (Figure 2A). Th-S counts at 0 h were approximately 2000 for both the α-syn sample incubated either alone or in the presence of EA. However, α-syn fibrils that were incubated alone continued to aggregate further over time, as evidenced by the increase in Th-S counts (Figure 2A), while the α-syn fibrils sample incubated in the presence of EA significantly disaggregated after 48 h, as indicated by the Th-S counts. We also observed that EA-mediated disaggregation occurred in a concentration-dependent manner, reaching its peak levels after 48 h with a 1:6 molar ratio (Figure 2A).

### 2.3. EA Interferes with the Seeding of α-Syn Monomers

By referring to the nucleation-dependent polymerization model, the formation of the amyloid fibril occurs in three steps [18]. The lag phase or nucleation phase will be the first step, where the soluble fraction will be nucleated and form oligomeric species, then the polymerization or growth phase starts as the oligomers polymerize to reach to final product of insoluble fibrils (equilibrium phase). Adding small preformed aggregates (seeds) will accelerate this process, bypassing the longest phase of fibril formation (nucleation phase) in vitro and in vivo through a process known as seeding. According to our results, we showed that EA has the ability to interfere with α-syn fibril formation (Figure 1) as well as disaggregate the preformed fibrils (Figure 2A). Thus, it was our interest to test if this compound has any effect on the seeding of α-syn aggregation. Preformed mature α-syn fibrils were briefly sonicated as described in the Materials and Methods Section (Section 4) to generate short fibrils (seeds), which were then added to the monomeric species to initiate aggregation. Samples of 100 µM of monomeric α-syn containing a final concentration of 2 µM of seeds were incubated with continuous shaking at 37 °C for 5 h, either alone or in the presence of EA at two different concentrations (10 or 50 µM), and the intensity of Th-s fluorescence emission was recorded every hour. Our results showed that the addition of seeds accelerates the α-syn aggregation process (Figure 2B). As anticipated, the addition of short fibrillar seeds enhanced the fibrillation process of the α-syn monomers, while the presence of EA at concentrations of 10 and 50 µM inhibited the seeding process significantly, as shown by the low Th-S intensity, indicating that EA interfered with the α-syn seeding process.

### 2.4. EA Prevents α-Syn Seed-Induced (Aggregated) Cytotoxicity in SH-SY5Y Cells

Human neuroblastoma cells (SH-SY5Y) expressing endogenous α-syn (Supplementary Figure S1) were treated either with α-syn alone (aggregated) or with EA, and incubated for 11 days at three different ratios. Cell death and viability was estimated using the MTT assay. To ensure the toxicity effect of EA on cells, and prior to any experiment, cell viability was assessed by using the different concentrations of EA and only less-toxic doses were later employed for the experiments with aged α-syn solutions (Supplementary Figure S2).

We observed that aged α-syn (5 µM) significantly inhibited the reduction of MTT (Figure 2C), and it is known that the reduction of MTT is directly proportional to the number of live cells. However, samples of α-syn incubated in the presence of EA (1:4, 1:2, and 1:1) were less toxic to the cells, as shown by the increased MTT reduction (Figure 2C), which indicates the number of surviving and live cells. At a concentration of 5 µM of α-syn aged alone, the induction of viable cells reduced by almost 75%, however in the presence of EA at a 1:4 ratio, the survival of the cells improved dramatically, reaching 60%. Interestingly, at a molar ratio of 1:4, EA was shown to be a very good inhibitor of fibril formation (Figure 1). These findings are in line with the Th-S fluorescence measurements (Figure 1A,B) and the immunoblotting analyses (Figure 1C), which clearly demonstrate that inhibition of α-syn aggregation by EA was comparable at a 1:4 molar ratio.

### 2.5. EA Prevents Aggregated α-Syn-Mediated Increase of Apoptotic Markers and Enhances the Levels of the Anti-Apoptotic BCL-2 in SH-SY5Y Cells

**Figure 3:**
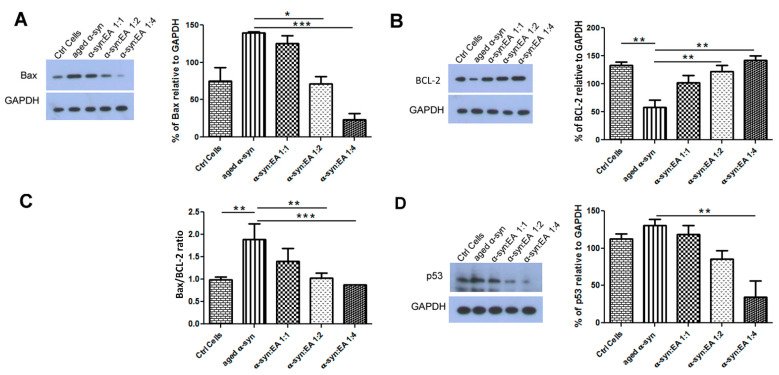
Expression of apoptotic markers, Bax, BCL-2, and tumor suppressor protein p53 in SH-SY5Y wt cells treated with α-syn alone or pre-incubated with different molar ratios of EA. SH-SY5Y wt neuronal cells were seeded in 24-well plates at a density of 5 × 10^4^ cells/well, and the cells were maintained for 24 h before treating for 24 h with a 5 µM final concentration of α-syn pre-incubated for 11 days at 37 °C with continuous shaking, in the presence or absence of EA at molar ratios of α-syn:EA of 1:1, 1:2, and 1:4. (**A**) Western blot analysis to examine the effect α-syn:EA on the expression of Bax in SH-SY5Y cells (**left** panel), and the results were quantified as percentage relative to GAPDH expression (**right** panel). (**B**) Western blot analysis to examine the effect α-syn:EA on the expression of BCL-2 in SH-SY5Y cells (**left** panel), and the results were quantified as percentage relative to GAPDH expression (**right** panel). (**C**) Ratio of the expression of Bax/BCL-2. (**D**) Western blot analysis to examine the effect α-syn:EA on the expression of the tumor suppressor protein p53 in SH-SY5Y cells (**left** panel), and the results were quantified as percentage relative to GAPDH expression (**right** panel). Data represent the mean value of three independent experiments ± standard deviation. Statistical analysis was performed using one-way ANOVA followed by the Bonferroni Multiple Comparison Test, ***, *p* < 0.001; **, *p* < 0.01; *, *p* < 0.05.

**Figure 4:**
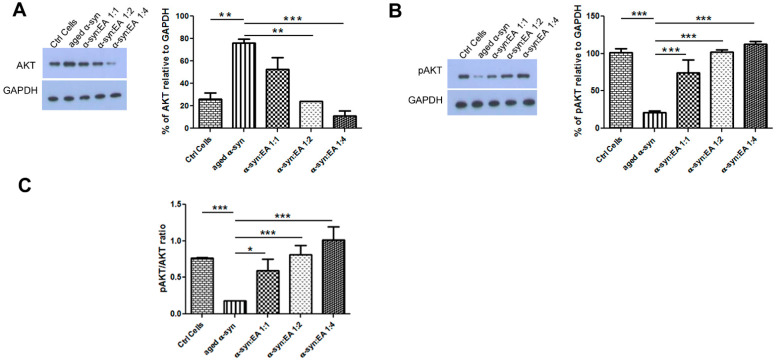
Expression of cell proliferation markers AKT/pAKT in SH-SY5Y wt cells treated with α-syn alone or α-syn pre-incubated with different molar ratios of EA. SH-SY5Y wt neuronal cells were seeded in 24-well plates at a density of 5 × 10^4^ cells/well, and the cells were maintained for 24 h before treating for 24 h with a 5 µM final concentration of α-syn pre-incubated for 11 days at 37 °C with continuous shaking, in the presence or absence of EA at molar ratios of α-syn:EA of 1:1, 1:2, and 1:4. (**A**) Western blot analysis to examine the effect α-syn:EA on the expression of AKT protein in SH-SY5Y cells (**left** panel), and the results were quantified as percentage relative to GAPDH expression (**right** panel). (**B**) Western blot analysis to examine the effect α-syn:EA on the expression of pAKT in SH-SY5Y cells (**left** panel), and the results were quantified as percentage relative to GAPDH expression (**right** panel). (**C**) Ratio of the expression of pAKT/AKT. Data represent the mean value of three independent experiments ± standard deviation. Statistical analysis was performed using one-way ANOVA followed by the Bonferroni Multiple Comparison Test, ***, *p* < 0.001; **, *p* < 0.01; *, *p* < 0.05.

**Figure 5:**
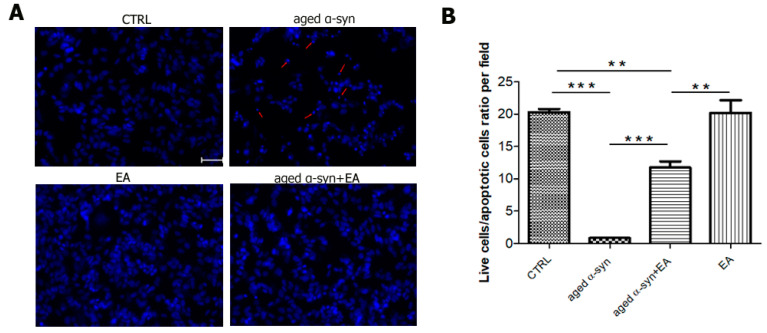
Aged α-syn causes nuclear condensation and DNA fragmentation in SH-SY5Y cells. SH-SY5Y cultured cells were treated with vehicle, vehicle + Ellagic acid, aged α-syn, and Ellagic acid + aged α-syn for 24 h, as indicated in the figure. (**A**) Cells were fixed, and stained with Hoechst 33342. Aged α-syn exposure significantly increased the apoptotic cells, as shown by the arrow. (**B**) Number of live and apoptotic cells are counted per field and presented as a ratio. Statistical analysis was performed using one-way ANOVA followed by the Bonferroni Multiple Comparison Test, ***, *p* < 0.001; **, *p* < 0.01. Scale bar 50 µm.

To test the neurotoxic mechanism of aggregated α-syn, we treated dopaminergic neuroblastoma SH-SY5Y cells with aggregated α-syn alone or pre-incubated α-syn with EA. We found that aggregated α-syn increased the apoptotic indicator BAX and decreased the anti-apoptotic indicator BCl-2, as expected. Interestingly, we found that EA incubated with α-syn not only prevented the aggregation of α-syn in the test tube, but also decreased BAX activation while enhancing BCL-2 levels (Figure 3A,B, respectively). The ratio of BAX to BCL-2 also indicated that EA treatment reduced this ratio (Figure 3C). This result suggests that EA blocks the aggregation of α-syn, which limits its toxic effects. We also investigated whether the apoptotic protein p53 plays a role in this cellular model. Interestingly, we found that aggregated α-syn also significantly enhanced p53 levels, whereas α-syn incubated with EA reduced the expression of p53 (Figure 3D). Additionally, we investigated the levels of AKT and pAKT—an active form of AKT. Aggregated α-syn increased the total AKT level, however it decreased the pAKT level. Interestingly, EA pre-treatment of α-syn reduced total AKT and increased pAKT. We also determined the ratio of pAKT to AKT and observed a similar trend (Figure 4). Since we observed that Ellagic acid modulated the aged α-syn-induced increased apoptotic marker Bax and decreased the ant-apoptotic marker BCL-2, we would like to examine if it also reduced the apoptotic cells. We performed the Hoechst staining and observed that EA pretreatment significantly reduced the number of apoptotic cells compared to only aged α-syn-treated cells (Figure 5A,B).

### 2.6. EA Modulates Autophagic Clearance of Aggregated α-Syn

**Figure 6:**
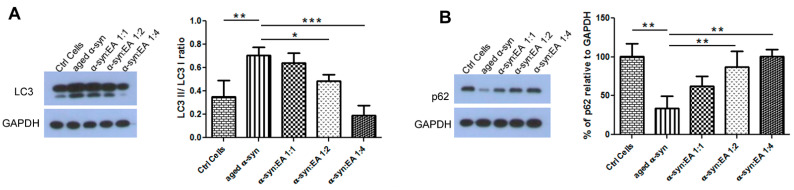
Expression of autophagy markers, LC3, and p62 proteins in SH-SY5Y wt cells treated with α-syn alone or α-syn pre-incubated with different molar ratios of Ellagic acid. SH-SY5Y wt neuronal cells were seeded in 24-well plates at a density of 5 × 10^4^ cells/well, and the cells were maintained for 24 h before treating for 24 h with a 5 µM final concentration of α-syn pre-incubated for 11 days at 37 °C with continuous shaking, in the presence or absence of EA at molar ratios of α-syn:EA of 1:1, 1:2, and 1:4. (**A**) Western blot analysis to examine the effect α-syn:EA on the expression of LC3 protein in SH-SY5Y cells (**left** panel), and the results were quantified as the ratio percentage of LC3-II/LC3-I relative to GAPDH expression (**right** panel). (**B**) Western blot analysis to examine the effect α-syn:EA on the expression of p62 protein in SH-SY5Y cells (**left** panel), and the results were quantified as percentage relative to GAPDH expression (**right** panel). Data represent the mean value of three independent experiments ± standard deviation. Statistical analysis was performed using one-way ANOVA followed by the Bonferroni Multiple Comparison Test, ***, *p* < 0.001; **, *p* < 0.01; *, *p* < 0.05.

Since the clearance of aggregated α-syn offers a promising strategy for the treatment of PD [19,20], earlier studies have shown that autophagy-mediated α-syn degradation favors a beneficial effect against PD [21,22]. We observed that aggregated α-syn increases the LC3-ll conversion from LC3-I. This result suggests the formation of autophagosomes for the clearance of aggregated α-syn. However, the clearance of autophagosomes containing misfolded or aggregated proteins requires p62 as an adaptor protein. We found that p62 levels significantly decreased when SH-SY5Y cells were treated with aggregated α-syn, indicating the failure or alteration in degradation of aggregated α-syn through lysosomes and lethal accumulation of autophagosomes [14,15,16,17,23,24,25]. However, incubation of α-syn with EA prevents the formation of aggregated α-syn and thus normalizes the impaired autophagy, as evidenced by suppression of LC3-II and enhancement of p62 expression (Figure 6), resulting in autophagosome maturation and neuroprotection, consistent with higher levels of pAKT [26,27].

**Figure 7:**
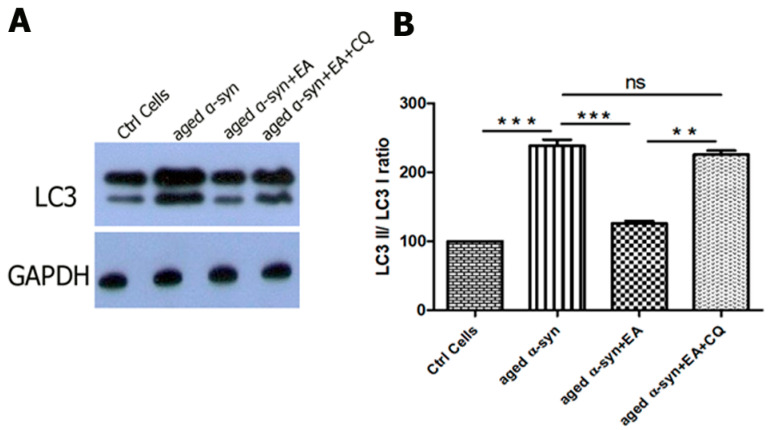
Improvement of autophagic flux by EA in α-syn-treated cells. Human neuroblastoma SH-SY5Y wt cells were seeded into 24-well plates and incubated for 24 h. The cells were treated with 20 µM of EA 3 h prior to 5 µM preformed α-syn fibrils exposure. To examine the autophagic flux, cells were treated with the autophagy inhibitor, chloroquine (10 µM), 1 h before α-syn fibrils exposure, as mentioned in the Methods Section. (**A**) Western blot analysis to examine the effect on the expression of LC3 protein in SH-SY5Y cells. (**B**) The results were quantified as percentage relative to GAPDH expression. Note that adding CQ increased the accumulation of LC3-II, indicating the activation of autophagic flux by EA. Data represent the mean value of three independent experiments ± standard deviation. Statistical analysis was performed using one-way ANOVA followed by the Bonferroni Multiple Comparison Test, ***, *p* < 0.001; **, *p* < 0.01, ns: non-significant.

Autophagic flux includes the sequential events of LC3-II-mediated autophagosomes’ formation, their fusion with lysosomes forming autosomes, and release and recycling of degraded macromolecules. This is called productive or completed autophagy. However, the increases in the levels of LC3-II are not measures of autophagic flux per se, as they may reflect the activation of autophagy or suppression of autophagosome clearance and accumulation of autophagosomes, which may result from failed fusion with lysosomes or lysosome dysfunction. This suppression of autophagosome clearance is called imperfect or altered autophagic flux, which may induce cell death. Autophagic flux can be monitored with autophagy inhibitors such as chloroquine (CQ), bafilomycin A1 (Baf A1), or lysosomal protease inhibitors. This can be determined by measuring LC3-II levels in both the presence and absence of inhibitors; if flux is occurring, the amount of LC3-II will be higher when the inhibitor is present [12,13,14]. We have observed the accumulation of LC3-II when CQ is added, as shown in Figure 7.

## 3. Discussion

PD is the second most common neurodegenerative disease after AD. The pathological hallmarks of the disease are selective loss of dopamine-producing neurons in the SNc area and accumulation of intracytoplasmic aggregated proteins known as LBs in surviving neurons. The major constituent of LBs is a protein with 140 amino acids called α-syn. The physiological functions of α-syn are not fully understood. However, it has been shown that α-syn impairs the release or re-uptake of neurotransmitters (dopamine) [28,29]. Interestingly, mutations in the α-syn gene, SNCA, have been identified in several families, thus highlighting the role of α-syn in PD progression.

Since α-syn protein is not only associated with the familial form of PD, but it is also related to sporadic PD, where it is the major contributor, research has been conducted to identify small molecules or natural compounds that can block or modulate the aggregation process of α-syn. Thus, targeting α-syn may be considered a vital therapeutic strategy against PD. Monomeric α-syn is natively unfolded in solution, and it has been reported that α-syn undergoes a process of self-association, leading to oligomerization when incubated at room temperature, and that aggregated α-syn species (oligomers) are toxic to cells [4,30,31]. Aggregated α-syn can affect many cellular pathways and cause neuronal death. Our study also found that aggregated α-syn causes cell death when added to the cell culture system. However, we found that EA co-incubated with α-syn prevented aggregation, and the mixture (α-syn:EA) was less toxic than aggregated α-syn (Figure 1A and Figure 2C, respectively). Consistent with this, EA has been shown to prevent SNO-PDI formation in a cellular model, which otherwise enhances α-syn aggregation, leading to cell death [7].

In the present study, we found that aggregated α-syn increased the protein levels of BAX and p53 and concomitantly decreased the anti-apoptotic marker BCL-2 in cultured cells, which could be related to dysfunctional autophagy, as mentioned above. However, EA co-incubated with α-syn decreased BAX and p53 and increased the anti-apoptotic protein BCL-2, which was consistent with the anti-apoptotic role of BCL-2 [25,32]. We also found that aggregated α-syn increased AKT, however, it decreased the active form of AKT (pAKT). Interestingly, the presence of EA with α-syn significantly increased pAKT, suggesting a beneficial role of EA in preventing α-syn-mediated toxicity, thus conferring cryoprotection [26,27]. A novel finding in the current study is the restoration of α-syn-induced impairment of autophagy in SH-SY5Y cells by EA. We observed that aggregated α-syn alters autophagic turnover via excessive autophagosome formation and accumulation, as indicated by an increase in the LC3-II/LC3-I ratio and a reduction in autophagic receptor p62 levels, suggesting impairment of clearance of aggregated α-syn by accumulated immature autophagosomes [17,23,24,25]. The addition of EA to α-syn normalized the ratio of LC3-II/LC3-I and dose-dependently increased the p62 level, which is essential for the maturation of autophagosomes and their clearance by lysosomes via the formation of autolysosomes, resulting in suppression of cell death [14,15,16,17,24,27]. In brief, our study shows novel findings related to the mechanisms of the neuroprotective effects of EA in α-syn-treated cells. These mechanisms are primarily linked to the restoration of altered autophagy through the normalization of p62 and LC3-II autophagy proteins, resulting in the suppression of cell death. These neuroprotective effects of EA may be related to autophagic degradation of intracellular α-syn. Additionally, and in association with autophagy restoration, EA improves the expression of anti-apoptotic factors such as BCL-2 and reduces apoptosis effectors’ levels. Further studies are underway in our laboratory to confirm these results in various in vivo animal models of Parkinson’s disease.

**Figure 8:**
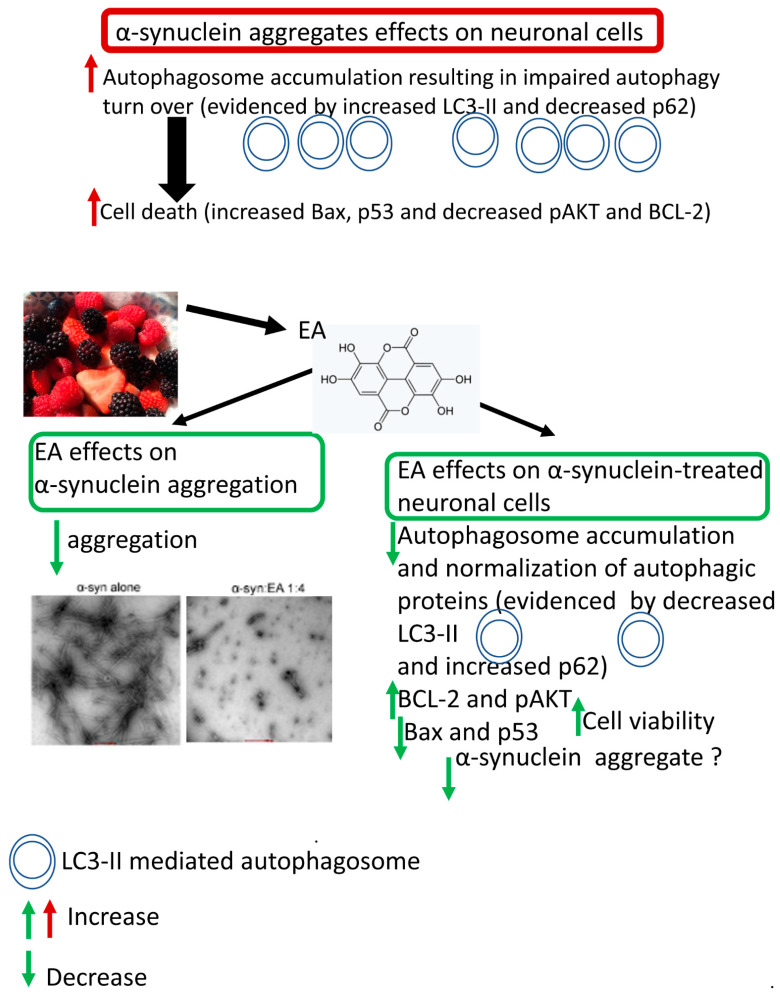
A schema showing the neuroprotective effects of EA in α-syn-treated cells. EA reduces aggregated α-syn in vitro. Mechanistically, α-syn induces autophagy impairment or dysfunction in treated cells, as evidenced by the reduction of p62 level and increased LC3-II-related accumulation of autophagosomes, resulting in enhanced apoptosis. On the other hand, EA reverses these effects of α-syn by normalization or restoration of autophagy and suppression of apoptosis. These neuroprotective effects of EA could be related to autophagic degradation of intracellular α-syn.

In conclusion, our current data suggest that α-syn aggregates induce apoptotic death in SH-SY5Y cells and EA mitigates α-syn-induced cellular toxicity by controlling α-syn aggregation. Aggregated α-syn increased apoptotic markers and reduced anti-apoptotic BCL-2 expression. However, pre-incubation of α-syn with EA reversed these effects. Moreover, aggregated α-syn induced autophagy dysfunction or impairment, resulting in cellular damage. In contrast, EA treatment restored autophagy by normalizing the levels of LC3-II, p62, and pAKT. The mechanism of α-syn aggregate-induced apoptosis is unclear, and the underlying mechanism of EA-mediated protection is not fully understood. However, we anticipate that the beneficial effects of EA are modulated by inhibiting aggregate formation from α-syn and restoring autophagy. Further studies are necessary to understand the various mechanisms underlying EA-induced neuroprotection in PD. A schema showing the summary of the results and conclusions of the present study is shown in Figure 8.

## 4. Materials and Methods

### 4.1. Bacterial Expression System for Purification of Recombinant Human α-Syn

We used the expression vector pT7-7 wt-α-syn to purify recombinant human α-syn, as reported earlier (pT7-7 wt-α-syn was a gift from Hilal Lashuel, Addgene plasmid # 36046; http://n2t.net/addgene:36046 (accessed on 12 November 2021); RRID: Addgene_36046) [33,34,35]. Briefly, using the bacterial *E. coli* strain BL-21 DE-3 cells, the expression of wt-α-syn was induced by the addition of isopropyl D–thiogalactopyranoside (IPTG). The cells were then collected and resuspended in 1× PBS containing 5 mM of EDTA, 0.02% sodium azide, and the pellets were then homogenized using a glass homogenizer and sonicated for 10 min. The homogenate was then boiled for 10 min and kept on ice for 30 min for cooling. The clear cell lysate was obtained by centrifugation at 15 K for 20 min. Prior to FPLC purification, the supernatant was subjected to dialysis against gel filtration buffer (10 mM Tris, pH 7.6, 50 mM NaCl, 1 mM EDTA, 1 mM PMSF), filtered through 0.22 um filters, and finally concentrated to the range of 2–4 mL/L of cell culture using protein concentration columns (MWCO 7 K, Thermo Fisher, Waltham, MA, USA). A Superdex 200 gel filtration column (GE Healthcare, Chicago, IL, USA) was used for PFLC purification, the selected fractions were pooled and examined by SDS, and the protein concentration was determined by the standard BCA assay.

### 4.2. In Vitro Aggregation of α-Syn

The aggregation of α-syn with or without EA was carried out as recently described by us [35]. Briefly, EA stock solutions (10 mM) were prepared using 100% ethanol. Samples of 25 µM α-syn protein in the presence or absence of different molar ratios of EA (α-syn:EA, 1:1, 1:2, and 1:4) were subjected to aggregation by incubating the samples for 11 days with continuous shaking (8000 rpm) at 37 °C using a thermomixer (Eppendorf, Hamburg, Germany). Formation of α-syn fibrils was detected by a thioflavin-S binding assay.

### 4.3. Thioflavin-S (Th-S) Assay

The fibril formation of α-syn was checked at different time points using the Th-S binding assay, as mentioned previously [35]. By interacting with fibrils containing a β-sheet structure, Th-S dye exhibits fluorescence resulting from binding to amyloid fibrils, which can be detected at excitation and emission wavelengths of 450 and 486. To monitor the effect of EA on α-syn aggregation, 10 µL of sample was added to 40 µL of Th-S (final concentration is 5 µM of α-syn and 20 µM of Th-S) in a 384-well, black micro-well plate (Nunc Cell Culture/Thermo Fisher Scientific, Waltham, MA, USA), and readings were taken using a microplate reader (Infinite M200PRO-TECAN, Männedorf, Switzerland).

### 4.4. Transmission Electron Microscopy

Samples from day 11 (α-syn:EA) (5 µL) were added to copper grids, 400-mesh (Agar Scientific, Essex, UK), as described previously [36,37]. The samples on the grids were then fixed by adding 5 µL of 0.5% glutaraldehyde, and then negatively stained with 2% uranyl acetate. Images were viewed using a Philips CM-10 transmission electron microscope.

### 4.5. Seeding Polymerization Assay

As described in [36,37], the seeding polymerization assay was carried out by fragmenting α-syn fibrils by sonication to obtain short fibrils (seeds), and 2 μM of the obtained seeds were added to monomeric α-syn (100 μM) and incubated in a thermomixer at 37 °C for 5 h in the presence or absence of 10 or 50 μM of EA with continuous shaking. The fibril formation in α-syn samples was monitored by the Th-S binding assay, as described above.

### 4.6. α-Syn Disaggregation Assays

The preformed α-syn aggregates at a concentration of 25 μM were incubated in the presence or absence of EA at molar ratios of α-syn:EA of 1:1, 1:4, and 1:6 for 48 h at 37 °C, with continuous mixing at 800 rpm. The fibril content was estimated by the Th-S assay at selected time points.

### 4.7. Culture Condition of SH-SY5Y Human Neuroblastoma Cells

Wild-type SH-SY5Y human neuroblastoma cells were procured from Addexbio (San Diego, CA, USA). Cells were grown and maintained in Dulbecco’s MEM/F-12 (1:1) (1×) (Gibco/Thermo Fisher Scientific, Waltham, MA, USA) containing 15% fetal bovine serum and 1% penicillin–streptomycin (P/S; 100 U/mL penicillin, 100 mg/mL streptomycin), L-glutamine, and 15 mM of HEPES. The cells were maintained at 37 °C in a humidified incubator with 5% CO_2_/95% air.

### 4.8. Cell Cytotoxicity (MTT) Assay

A density of 15,000 SH-SY5Y cells suspended in DMEM F-12 (1:1) medium were plated in a 96-well plate (200 µL/well). After 24 h of incubation, the medium was replaced with 200 μL of serum-free OPTI-MEM medium (Gibco/Thermo Fisher, Waltham, MA, USA) containing α-syn aged with or without EA at molar ratios of 1:1, 1:2, and 1:4. Briefly, α-syn alone or different molar ratios of α-syn with EA were incubated as mentioned above to induce aggregation of syn for 11 days. The reaction mixtures were collected and used in the MTT assay to evaluate the cellular toxicity. The treated cells were then incubated at 37 °C in 5% CO_2_ for 48 h. A final concentration of MTT (0.5 mg/mL) in PBS was added to each well and the plate was re-incubated again at 37 °C for 4.5 h. At the end of incubation, the medium was removed carefully, and 100 μL of lysis buffer (15% SDS, 50% N, *n*-dimethylformamide, pH 4.7) was added. The plates were incubated overnight at 37 °C. Absorbance values at 560 nm were recorded using a microplate reader (Infinite M200PRO, TECAN, Männedorf, Switzerland).

### 4.9. Western Blot Analysis of Apoptotic, Anti-Apoptotic Proteins, and Autophagic Indicators

A density of 100,000 SH-SY5Y cells were suspended in DMEM F-12 (1:1) medium and seeded in a 12-well plate (1000 µL/well). After 24 h of incubation, the medium was replaced with 1 mL of serum-free OPTI-MEM medium (Gibco) containing 5 µM of aggregated α-syn aged with or without EA at molar ratios of 1:1, 1:2, and 1:4. Twenty-four hours after incubation, the media was removed, and cells were washed with 1× PBS, harvested, and used for protein extraction using 1× RIPA buffer (Millipore, USA, Cat number 20–188). Proteins were quantified using the BCA method. Twenty micrograms of proteins were separated by SDS-PAGE and transferred to PVDF membranes using an electrophoretic transfer system (Bio-Rad, Hercules, CA, USA). The membranes were blocked with 5% non-fat dry milk in 1× PBS containing 0.1% Tween 20 (PBS-T) for 1 h, followed by incubation with primary antibodies against BCL-2 (1:1000, Santa Cruz Biotechnology, Dallas, TX, USA. Cat# sc-7382), BAX (1:1000, Cell Signaling Technology, Danvers, MA, USA, Cat# 2772S), p53 (1:1000, Cell Signaling Technology, Danvers, MA, USA, Cat# 2524S), AKT (1:1000, Cell Signaling Technology, Danvers, MA, USA, Cat# 2920S), LC3 (1:1000, Cell Signaling Technology, Danvers, MA, USA, Cat# 12741S), p62 (1:1000, Abcam, Cat# ab56416), and GAPDH (1:1000, Cell Signaling Technology, Danvers, MA, USA, Cat# 2118S), overnight at 4 °C. For pAKT (1:1000, Cell Signaling Technology, Danvers, MA, USA, Cat# 9271S), the membrane was blocked with 5% BSA instead of 10% non-fat milk. The membranes were then incubated with the corresponding secondary antibody at room temperature for 1 h.

To examine the autophagic flux, cells were treated with EA (20 µM) 3 h before the aged α-syn (5 µM) treatment. The autophagy inhibitor, chloroquine (CQ; 10 µM), was added 1 h before the treatment of aged α-syn and further incubated for 24 h. The cells were then harvested and lysed by 1× RIPA buffer and the total protein estimation was carried out by the BCA assay. Ten µg of the cell lysate was loaded into 15% SDS gel and the transferred membrane was probed with anti-LC3 and GAPDH antibodies.

### 4.10. Detection of Apoptotic Cells Using Nuclear Staining (Hoechst 33342)

Chromosomal condensation and morphological changes in the nucleus are considered as markers of apoptosis. We have used the chromatin dye, Hoechst 33342, to stain SH-SY5Y wt cells to evaluate nuclear condensation. The SH-SY5Y cells were seeded on 4-well plates and incubated for 24 h. The cells were then treated with aged α-syn or pretreated with EA three hours before treatment of aged α-syn or EA alone. The cells were incubated further for 24 h. The cells were then fixed with 4% paraformaldehyde in 0.1 M phosphate-buffered saline (PBS) for 20 min. After 3 washes with the PBS buffer, cells were stained with Hoechst 33342 (ThermoFisher Scientific, Waltham, MA, USA) for 5 min. Cells were then washed three times with PBS and kept in the same buffer solution. Cells were analyzed under an inverted fluorescence microscope (Ziess, Axiovert 40 CFL; Oberkochen, Germany) and images were taken from different groups for further analysis. The number of apoptotic cells (condensed and fragmented nuclei) as well as live cells was counted from each group using a similar field (80 mm^2^). The data were presented as ratio of live to apoptotic cells per field.

### 4.11. Protein Estimation

The protein content was estimated using the Pierce BCA Protein Assay Kit (Thermo Scientific, Waltham, MA, USA) following the manufacturer’s instructions.

### 4.12. Statistical Analyses

Data are expressed as the mean ± standard deviation. The data for all studies were analyzed with GraphPad Prism software v5.0 (GraphPad Software, San Diego, CA, USA) using one-way ANOVA followed by the Bonferroni Multiple Comparison Test. Asterisks represent significant differences: ***, *p* < 0.001; **, *p* < 0.01; *, *p* < 0.05.

## Data Availability

The authors declare that the data supporting the findings of the current study are available in the manuscript.

## Supplementary Materials

The following are available online at https://www.mdpi.com/article/10.3390/ijms222413398/s1.
