# Supplementary material for: Ellagic Acid Prevents α-Synuclein Aggregation and Protects SH-SY5Y Cells from Aggregated α-Synuclein-Induced Toxicity via Suppression of Apoptosis and Activation of Autophagy
Source: Int J Mol Sci. 2021 Dec 13;22(24):13398. doi: 10.3390/ijms222413398 (PMC8707649; doi:10.3390/ijms222413398)
Supplement: Supplementary file 1 [file ijms-22-13398-s001.zip › ijms-1483057-supplementary.pdf]

International Journal of
Molecular Sciences

Supplementary Materials

Supplementary Figure S1

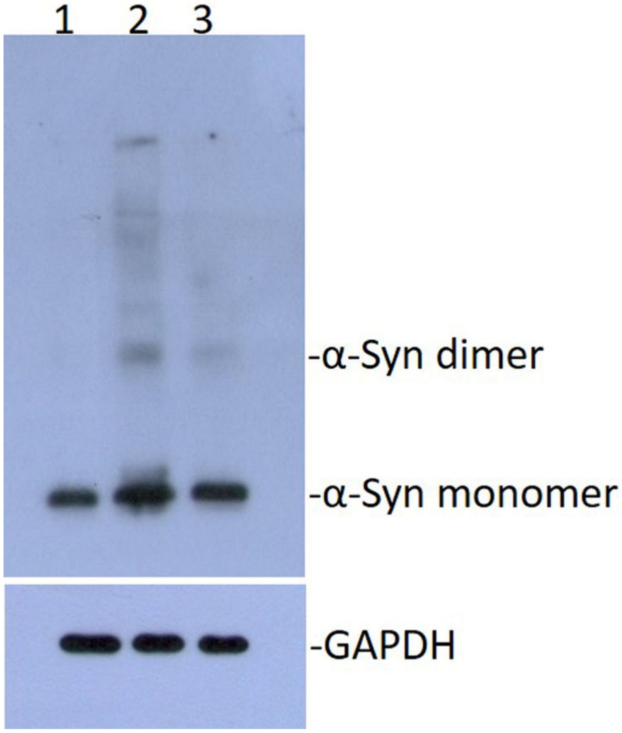

Figure S1. Effect of EA on the endogenous α-syn in human neuroblastoma SH-SY5Y cells. Cells were seeded in 24-well plate and maintained for 24 hours. (lane 1) CTRL cells, (lane 2) cells treated with 5μM preformed α-syn fibrils, (lane 3) cells treated with 5 μM preformed α-syn fibrils+20 μM EA. EA was added to cells 3 hours prior treatment with the preformed fibrils. Cells were incubated 24 hours after treatment, and then cells were lysed by 1× RIPA buffer, and the total protein was estimated by the BCA assay. 10 μg of the cell lysate was loaded into 15% SDS gel and the transferred membrane was probed with anti-human α-syn monoclonal antibody Syn1 (BD, USA) and GAPDH antibodies.

Supplementary Figure S2

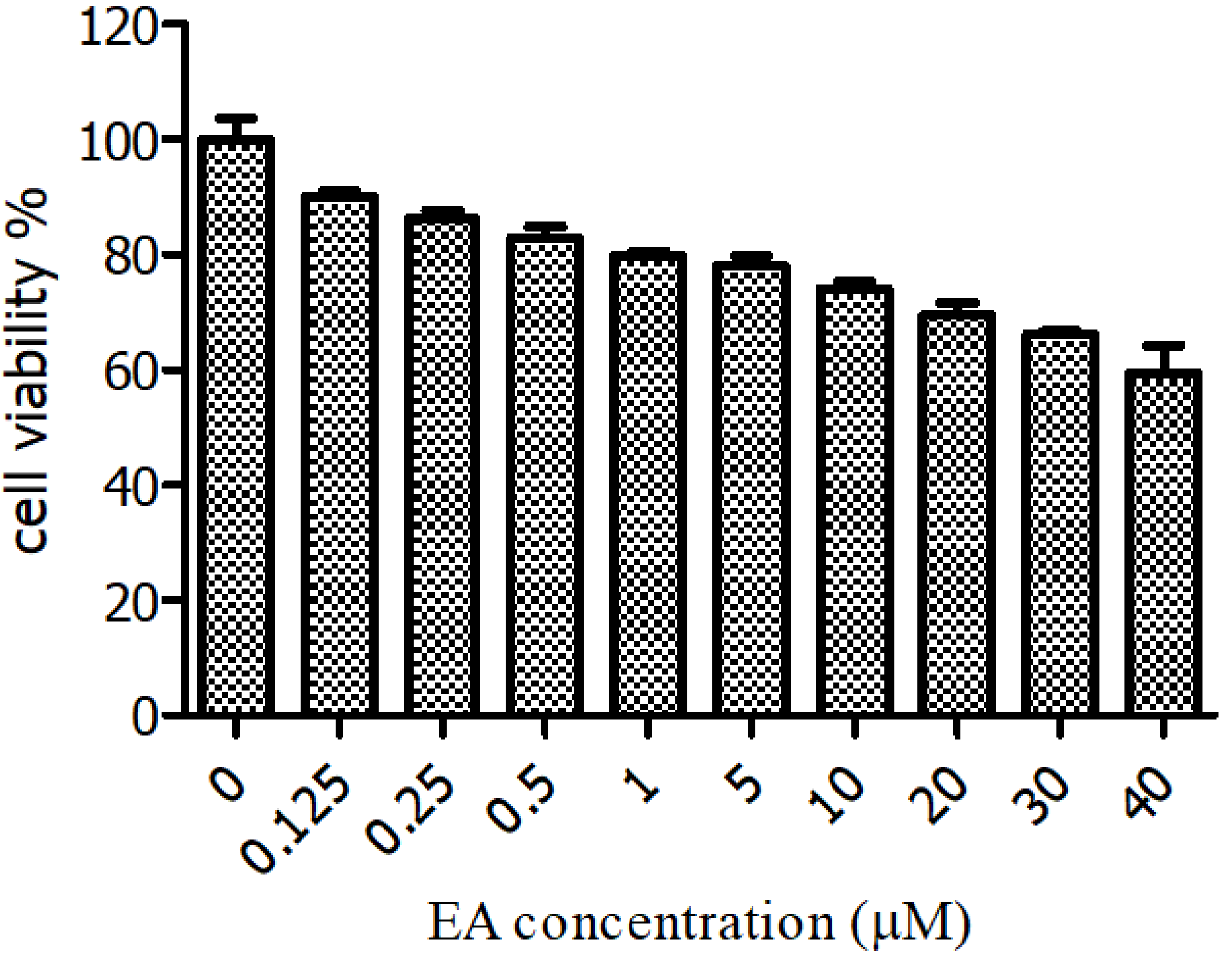

Figure S2. The MTT assay was performed to assess the cytotoxicity of ellagic acid (EA) on SH-SY5Y human neuroblastoma cells. The cells were treated for 48 hours prior to MTT addition with different concentrations of EA (0–40 μM). The results are expressed as percentages of the control (i.e., untreated cells) as an average value ± standard deviation.
